# Measuring Engagement in eHealth and mHealth Behavior Change Interventions: Viewpoint of Methodologies

**DOI:** 10.2196/jmir.9397

**Published:** 2018-11-16

**Authors:** Camille E Short, Ann DeSmet, Catherine Woods, Susan L Williams, Carol Maher, Anouk Middelweerd, Andre Matthias Müller, Petra A Wark, Corneel Vandelanotte, Louise Poppe, Melanie D Hingle, Rik Crutzen

**Affiliations:** 1 Freemasons Foundation Centre for Men's Health School of Medicine University of Adelaide Adelaide Australia; 2 Department of Movement and Sports Sciences Ghent University Brussels Belgium; 3 Health Research Institute, Centre for Physical Activity and Health Department of Physical Education and Sport Sciences University of Limerick Limerick Ireland; 4 Physical Activity Research Group, Appleton Institute School of Health, Medical and Applied Sciences Central Queensland University Rockhampton Australia; 5 Alliance for Research in Exercise, Nutrition and Activity, Sansom Institute School of Health Sciences University of South Australia Adelaide Australia; 6 Department of Rheumatology Erasmus Medical Center Rotterdam Netherlands; 7 Saw Swee Hock School of Public Health National University of Singapore Singapore Singapore; 8 Centre for Sport and Exercise Sciences University of Malaya Kuala Lumpur Malaysia; 9 Centre for Innovative Research Across the Life Course Faculty of Health and Life Sciences Coventry University Coventry United Kingdom; 10 Department of Nutritional Sciences College of Agriculture & Life Sciences University of Arizona Tucson, AZ United States; 11 Department of Health Promotion Care and Public Health Research Institute Maastricht University Maastricht Netherlands

**Keywords:** telemedicine, internet, health promotion, evaluation studies, treatment adherence and compliance, outcome and process assessment (health care)

## Abstract

Engagement in electronic health (eHealth) and mobile health (mHealth) behavior change interventions is thought to be important for intervention effectiveness, though what constitutes engagement and how it enhances efficacy has been somewhat unclear in the literature. Recently published detailed definitions and conceptual models of engagement have helped to build consensus around a definition of engagement and improve our understanding of how engagement may influence effectiveness. This work has helped to establish a clearer research agenda. However, to test the hypotheses generated by the conceptual modules, we need to know how to measure engagement in a valid and reliable way. The aim of this viewpoint is to provide an overview of engagement measurement options that can be employed in eHealth and mHealth behavior change intervention evaluations, discuss methodological considerations, and provide direction for future research. To identify measures, we used snowball sampling, starting from systematic reviews of engagement research as well as those utilized in studies known to the authors. A wide range of methods to measure engagement were identified, including qualitative measures, self-report questionnaires, ecological momentary assessments, system usage data, sensor data, social media data, and psychophysiological measures. Each measurement method is appraised and examples are provided to illustrate possible use in eHealth and mHealth behavior change research. Recommendations for future research are provided, based on the limitations of current methods and the heavy reliance on system usage data as the sole assessment of engagement. The validation and adoption of a wider range of engagement measurements and their thoughtful application to the study of engagement are encouraged.

## Introduction

Electronic health (eHealth) and mobile health (mHealth) behavioral interventions offer wide-reaching support at a low cost, while retaining the capacity to provide comprehensive, ongoing, tailored, and interactive support necessary for improving public health [1,2]. Although there is evidence that eHealth and mHealth behavior change interventions can be effective, low levels of adherence and high levels of attrition have been commonly reported [1-3]. In response, there have been calls to design and implement more engaging interventions to address these concerns [4-6].

It is generally agreed that a certain level of engagement is necessary for intervention effectiveness. However, there is a lack of clarity on how to conceptualize engagement. Some researchers have defined engagement solely as a psychological process relating to user perceptions and experience, whereas others consider engagement a purely behavioral construct, synonymous with intervention usage [4,7]. Consequently, it is often confused with adherence, which refers to whether the intervention is used as intended by the developers [3,8,9]. There have also been interdisciplinary differences. Behavioral scientists tend to characterize *good* engagement as high acceptability, satisfaction, or intervention adherence, whereas computer scientists tend to consider high engagement as a mental state associated with increased attention and enjoyment [4]. To consolidate these viewpoints and provide a less fragmented foundation for future research, 2 new conceptual models of engagement have been proposed [4,5].

Using a process of expert consensus, Yardley et al [5] proposed distinguishing between micro- and macrolevel engagement when examining the relationships between the user experience, usage, and behavior change. Microlevel engagement refers to the moment-to-moment engagement with the intervention, including the extent of use of the intervention (eg, number of activities completed) and the user experience (eg, level of user interest and attention when completing activities). Macrolevel engagement is defined as the depth of involvement with the behavior change process (eg, extent of motivation for changing behavior) and is linked to the behavioral goals of the intervention. The timing and relationship between micro and macro forms of engagement depend on the intervention, the user, and the broader context. Yardley’s model suggests that after a period of *effective* engagement at the microlevel, the user may disengage from the platform but still be immersed in the behavior change process. Perski et al [4] offer a similar but more extensive framework based on a systematic review. Similar to Yardley et al, they define engagement as both the extent of usage and a subjective experience but refine this further by characterizing the subjective experience as being related specifically to attention, interest, and affect. These constructs are said to capture the cognitive and emotional aspects of engagement as they are described in computer science disciplines (eg, flow, immersion, and presence), all of which relate to a level of absorption and preoccupation (see Table 1 for definitions of these constructs). According to Perksi et al [4], high engagement influences behavior change through its influence on the determinants of behavior (similar to macroengagement, as described by Yardley et al). Engagement itself is hypothesized to be influenced by intervention features such as content, mode of delivery, and contextual features such as the physical environment (eg, internet access) and individual characteristics (eg, internet self-efficacy).

Both Perski et al and Yardley et al extend previous models [6,9-11] by considering the interaction between usage and psychological processes. By doing so, both models suggest that intervention usage may be a useful indicator of overall engagement with the intervention but is not a valid indicator of engagement in the behavior change process per se. Perski et al also highlight potential moderators and mediators of the engagement process and outline possible pathways in which engagement can influence overall intervention efficacy. These models serve as useful tools to refine and test hypotheses about how to influence engagement and how engagement impacts efficacy, which is necessary if we are to advance eHealth and mHealth behavioral science. However, an understanding of how to measure engagement is needed to test these models.

Basic overviews of the types of measures to assess engagement in eHealth and mHealth interventions have been provided by Yardley et al [5] as well as Perski et al [4]. Yardley et al briefly described the potential usefulness of different measurement types, including qualitative measures, self-report questionnaires, ecological momentary assessment, system usage data, sensor data, and psychophysiological measures. Perski et al identified over 100 studies related to engagement and noted the data collection methods used (eg, survey, website logs, and face-to-face interviews) in each study. Our aim is to extend their work by providing a comprehensive overview of the measurement options currently available. Our overall goal is to summarize and appraise measures of engagement used in eHealth and mHealth research and to highlight future areas of research when evaluating engagement in eHealth and mHealth behavior change interventions. We anticipate this will serve as a useful primer for those interested in the study of engagement and help to advance the field of eHealth and mHealth and behavior change by facilitating the use and validation of a wider range of engagement measurements and their thoughtful application to the study of engagement.

## Overview of Methods Used to Identify and Assess Engagement Measures

We used a snowballing approach to identify relevant engagement measures. To begin, we extracted measures identified by Perski et al [4] as well as other systematic reviews and published articles known to us through our former work in the field [12-14]. A data extraction table (see Multimedia Appendix 1) focusing on measurement type, engagement domain, and validity information was used to extract, sort, and explore measurement information to aid synthesis. During the writing and revision process, we searched for additional articles using Google Scholar and reran Perski’s [4] original search strategy on MEDLINE and PsycINFO to identify more recent relevant literature. Readers should, therefore, consider this as a comprehensive, but not exhaustive, overview of the literature.

In line with Yardley et al’s suggestions [5], our overview focuses on a wide range of methods to measure engagement. These include qualitative measures, self-report questionnaires, ecological momentary assessment, psychophysiological measures, as well as the analysis of system usage data, sensor data, and social media data. Methods that capture microlevel constructs were included in our synthesis if they were related to emotional, cognitive, or behavioral aspects of the user experience that could be characterized as interest, attention, affect, or intervention usage. This includes the constructs of flow, cognitive absorption, presence, and immersion, which have been commonly used in other disciplines. An overview of definitions for each of these constructs is provided in Table 1. Macrolevel measures were included if they related specifically to engagement in the behavior change process because of the digital intervention or its features. A single author initially drafted each section below, with all other authors providing a critical review.

**Table 1:** Definitions for constructs used to describe the emotional, cognitive, or behavioral aspects of engagement in previous literature.

Construct	Description
Interest	Individual interest is an enduring preference for certain topics and activities. It is impacted by pre-existing knowledge, personal experiences, and emotions. Situational interest is an emotional state brought about by situational stimuli (eg, the unexpectedness of information). It is evoked spontaneously and is presumed to be transitory. Both types of interest are related to liking and willful engagement in a cognitive activity that affects the use of specific learning strategies and how we allocate attention [15,16].
Attention	A state of focused awareness of specific perceptual information [17]. Focalization and concentration of consciousness are the essence of attention. Paying attention implies withdrawal from some perceptual information to deal effectively with others [18].
Affect	Affect is an intrinsic part of the sensory experience. It represents how an object or situation impacts how a person feels. It can be described by 2 psychological properties: hedonic valence (pleasure/displeasure) and arousal (activation/sleepy). It can be a central or background feature of consciousness, depending on where and how attention is applied [19,20].
Flow	Flow refers to an optimal state that arises when an individual is deeply absorbed in a task. It is characterized by enjoyment, focused attention, absorption, and distorted time perception and is considered intrinsically rewarding. It assumes the complete absence of negative affect [21].
Cognitive absorption	Cognitive absorption is a state of deep involvement, similar to flow, though it does not assume intrinsic motivation or the complete absence of negative affect. Cognitive absorption may still occur when a user is frustrated (and, therefore, the experience is not optimal) or extrinsically motivated (eg, by winning a competition with friends; [22]).
Immersion	Immersion is also similar to cognitive absorption and flow, though it is often used to describe a less extreme experience of engagement, one where one may still have some awareness of one’s surroundings [22,23].
Presence	The term presence has been popular since the development of virtual reality technologies. Definitional consensus for presence is still emerging, though it is often described as the psychological sense of being *there* [24].
Intervention usage	The extent to which the intervention has been observed or interacted with by the user. It is made up of several components, including frequency of use, time spent on the intervention, and the type of interaction participated in. This is distinct from intended usage, which is the way in which users should utilize the intervention to derive the minimum benefit, as defined by the intervention developers [3].

## Overview of Engagement Measures

### Qualitative Methods

#### Focus Areas

Qualitative measures enable evaluation of micro- and macrolevel engagement and include methods such as focus groups, observations, interviews, and think-aloud activities (Table 2). At the microlevel, they allow for an in-depth account of the users’ experience of the intervention. At the macrolevel, they can be used to explore the users’ perceptions of how the intervention has helped them to engage in the behavior change process.

#### Current Use and Future Directions

Qualitative methodologies are commonly employed in the digital health setting to inform the development of interventions (ie, usability testing) and as an evaluation measure (eg, [25-29]). In most cases, the focus of the evaluation has been on perceptions of usability and acceptability, rather than engagement. However, there are some notable exceptions. For example, some studies have used think-aloud measures to understand cognitive processes and emotional reactions when navigating the intervention and viewing intervention content in real time [30-33]. Others have explored users’ flow experiences, adherence and lived experience of technology using qualitative interviews [34-36], focus groups [37], or a combination of think-aloud and interview methods [32].

Along with exploring the direct user experience, qualitative measures are also often used to probe the perceived usefulness of the intervention experience. Although this can relate to macroengagement (eg, by providing insights into how the intervention may have helped the user to achieve behavioral goals), efforts to explore the users’ experience of the behavior change process in more depth are recommended. For example, researchers could explore how certain intervention features impact intentions and self-efficacy and how the relationship between intervention features and changes in psychosocial factors relate to use or disuse. This could be achieved using simple methods such as open-ended items in a questionnaire or more elaborate methods such as postintervention focus groups, which may help users to reflect on how the intervention has or has not engaged them in the behavior change process in more detail. Assessing these constructs at different time points may be particularly fruitful, especially given the cyclical nature of behavior change [38]. Exploring users’ real-time engagement in the behavior change process was achieved in 1 recent study by thematically analyzing participant responses to intervention text messages [39]. By doing so, the authors were able to demonstrate that the study participants frequently gained positive cognitive and behavioral benefits from the text messages.

#### Considerations

A limitation of qualitative measures is that the results can be difficult to compare between studies. Results are also often not generalizable, mostly due to sampling bias. Qualitative measures are often used to collect rich data rather than representative data. For this reason, qualitative methods may be particularly suited to help generate hypotheses about engagement including how engagement relates to efficacy and effectiveness. They may also be useful for exploring hypotheses, especially when the focus is on understanding engagement on an individual level such as in n-of-1 studies [40]. In instances where representative data can be collected, such as in the text messaging study described above [39], hypothesis testing at the group level may be possible. However, the time and expertise needed to analyze data, which would ideally involve more than 1 person, is a barrier. This may be overcome in the future using machine learning tools to automate the coding of qualitative data [41].

**Table 2:** Overview of qualitative approaches to assessing engagement with considerations and example questions.

Qualitative approach	Description	Example items	Considerations (pros/cons)
Semistructured interviews	Provide an opportunity for sharing of lived experiences and feelings to uncover concealed perceptions related to digital health intervention or the technology; includes informal conversational interviews (spontaneous-suited to ethnographic research), semistructured interviews (interview guide used to steer otherwise spontaneous conversation), or standardized open-ended interviews (worded questions used for all participants).	Microlevel: Tell us what you think about the content; How did completing that module make you feel?; Please explain your pattern of use?; Why did you log on when you did?; Macrolevel: Did you notice any change to your thinking as a result of using the …(“app”)?; What impact did using the ... (“website”) have on how you are going about changing your behavior?	Pros: inform modifications to increase acceptability, interactivity and tailor to end-user needs; identify a range of issues associated with use (both short and long term); augment interpretation of quantitative evaluation; generally small sample sizes. Cons: subject to bias (eg, recall and social desirability), especially if leading questions are asked; time consuming to collect and transcribe; time consuming to analyze and often requires more than 1 person to decide on and confirm themes.
Think aloud	Aim to capture the experience of using the technology in real time. The user is provided with a specific task to complete and is observed while they perform the task. The user is prompted to think aloud throughout the process.	Microlevel: Tell me what you are thinking; What are you looking at?; What’s on your mind?; How are you feeling?; Why did you click on this?; Why did you frown/smile/sigh?; Macrolevel: Are you learning anything new?	Pros: can be used at various stages of development and implementation to understand how intervention features impact on engagement; occurs in real time, so less subject to recall bias. Cons: subject to observer bias; can be cognitively difficult for participants and requires practice; may require additional resources such as video or sound recording equipment to obtain a comprehensive picture. Acquired data can be time consuming and complex to analyze; may be most useful for exploring microlevel engagement.
Focus groups	Used to identify the social and contextual factors in specific population subgroups that influence engagement with digital health intervention and needs for technological characteristics and operations that promote user alignment and functional utility.	Microlevel: What did you think of the intervention?; Which components caught your attention the most?; What about them caught your attention?; Were there any components that caused frustration?; Did any aspects make you feel guilty? Macrolevel: How often did you think of the intervention during the week?; Was the intervention in the back of your mind?; How did the intervention help or hinder you reach your goals?	Pros: allow for spontaneous discussion of topics and subsequent voicing of ideas and perceptions that may go unnoticed in semistructured or structured interviews; Can obtain rich data from multiple people at the same time. Cons: subject to group or social desirability bias; some participants may not express themselves as fully in a group situation; requires practice to manage group discussion; can take a long time to transcribe due to interruptions/butting in; time consuming to analyze and often requires more than 1 person to decide on and confirm themes.

To facilitate the use of qualitative measures in the future, a brief overview of example questions by qualitative method type, as well as key considerations are provided in Table 2.

### Self-Report Questionnaires

#### Focus Areas

Questionnaires can be used to assess both experiential and behavioral aspects of microlevel engagement as well as aspects of macrolevel engagement.

#### Current Use and Future Directions

Self-report questionnaires have most often been used to gain insight into users’ subjective experience of digital platforms. Although questionnaire items have often been purpose-built and not subjected to psychometric testing (see Multimedia Appendix 1), there are a number of more rigorously developed scales. An overview of scales identified by our search [4,12-14] is presented in Multimedia Appendix 2. In brief, most scales have been developed to assess subjective experiential engagement with e-commerce websites or video games. Only 2 scales developed specifically for the eHealth and mHealth setting were identified (ie, the eHealth Engagement Scale [42] and the Digital Behavior Change Intervention Engagement Scale [43]), and only 1 of these has been validated [42], whereas validation of the other is currently underway [43]. Of note, some of the available scales assess attributes posited to predict engagement (eg, aesthetic appeal and usability experience [44-46]) as well as attributes considered to be a part of engagement (interest, attention, and affect). This is particularly the case for scales developed in the e-commerce setting and raises some validity concerns. Several of the scales are also quite long, which may place an undue burden on participants. The development and evaluation of high-quality short questionnaires relevant to eHealth and mHealth are therefore encouraged.

Questionnaires have also been used to assess behavioral aspects of engagement (ie, intervention usage). Although objective behavioral data are often available (see usage data below), questionnaires have been used when this is not the case. For example, a study comparing the relative efficacy of 2 *off-the-shelf* apps used questionnaires to assess the frequency and time of app use [47]. Although there are several scales with reasonable psychometric properties available for assessing the users’ subjective experience (Multimedia Appendix 2), scales for assessing behavioral aspects of engagement in eHealth and mHealth interventions are lacking. Perski et al’s self-report measure [43], which includes 2 items on behavioral engagement, is an exception. However, the validity of the measure is still being investigated. Perski’s items and the purpose-built item used by other researchers usually have reasonable face-validity (eg, “how many times per week did you use the app?”) but might lead to over- or underreporting depending on how items are phrased [48,49]. The validity of the chosen scale should be considered when interpreting the findings of self-reported behavioral data, and we recommend efforts to test the psychometric properties of developed items before use, if not yet available. This could be achieved by comparing the self-reported data with objectively collected data in a controlled setting (eg, [43]). The development of self-reported usage questionnaires that complement and provide useful context for objective usage measures should be considered. For example, if time on site or using an app is of interest, questionnaire data may identify cases where the user has left the program running in the background but has not been actively engaged. Likewise, information on behavioral cues at the point of engagement (eg, “what were you doing before you logged your steps using the app?”) may complement usage data and provide a more comprehensive measure of usage patterns. Lessons may be gleaned from the scales developed to assess social networking intensity [50].

The third use of questionnaires relevant to the study of engagement at the macrolevel is the repeated assessment of psychological mechanisms hypothesized to account for behavioral changes (eg, self-efficacy). The assessment of change in these mechanisms and the conduction of a formal mediation analysis have been increasingly encouraged in the behavioral sciences [51,52] to investigate whether interventions are working as intended (ie, that the selected eHealth and mHealth strategies are indeed influencing determinants and changes in determinants are influencing behavior, eg, [53]). This methodology can be adopted to study engagement. Arguably, a user who demonstrates favorable changes in 1 or more of these determinants can be considered engaged in the behavior change process (eg, self-efficacy significantly increases over time). Furthermore, someone demonstrating changes at a prespecified cut point or where changes are associated with behavioral outcomes could be said to be engaged effectively. There are a number of pre-existing scales that can be used to assess changes in psychological determinants of behavior (eg, [54-56]) as well as guides for constructing purpose-built questions if existing scales are not suitable (eg, [57,58]). Decisions regarding what psychological constructs to assess changes in should be based on the theoretical underpinning of the intervention and the key intervention objectives and strategies used to achieve them.

#### Considerations

Overall, questionnaires can be a useful tool for measuring various aspects of engagement in a systematic, standardized, and convenient way. This can allow for easy comparison across studies and between experimental arms [5]. Limitations include questionnaire length (and, therefore, duration of completion); a lack of experiential measures designed and tested within a health context; a lack of focus on the behavioral aspects of engagement; and in some cases, the inclusion of items that measure predictors of engagement within engagement scales.

To select an appropriate scale, an understanding of the different constructs used to describe engagement across disciplines will be necessary (see Table 1). Reviewing the wording of the items and assessing how they will fit within the context of one’s project may further help with scale selection. To this end, example items for each scale summarized above are provided in Multimedia Appendix 3. Most items will need to be adapted for a health setting, and not all scales will be applicable across study types or useful for assessing all aspects of engagement (ie, interest, attention, affect, intervention usage, and involvement in behavior change process). In some cases, it may be necessary to generate completely new items or a completely new scale. In such cases, researchers are encouraged to report a measure of internal consistency (preferably McDonald omega) and present factor-analytic evidence confirming the dimensionality of the scale [59]. Attention to the length of the scale should also be given. This will likely be necessary to minimize missing data. The perceptions of those who drop out of the study are currently often not captured in evaluations of eHealth and mHealth interventions, which is problematic as those who drop out are usually those who have used the intervention the least. Ecological momentary assessments (EMAs; described in more detail below) may be useful to assess relevant engagement parameters regularly during the intervention and give a better impression of engagement throughout use [60]. Alternatively, selecting a representative subsample to administer surveys to and reimbursing them for their time might be a viable solution.

### Ecological Momentary Assessments

#### Focus Area

EMAs can be used to assess both experiential and behavioral aspects of microlevel engagement as well as aspects of macrolevel engagement. The main objective of EMAs is to assess behaviors, perceptions, or experiences in real time and as they occur in their natural setting [61]. By prompting users to self-report data at varying times per day, EMAs allow these phenomena to be studied in different contexts and times.

#### Current Use and Future Directions

In EMAs, short surveys can either be accessed by the user on demand (eg, when logging a recent behavior), sent at specific or random intervals (eg, every 2 hours per day: *time-based sampling*), or they can be triggered by a certain event (eg, only when an activity tracker indicates the user is performing moderate to vigorous physical activity: *event-based sampling*). The latter is especially useful to capture rare behaviors, perceptions, or experiences. EMAs are often conducted on smartphone screens, but wearable devices can also be used (eg, CamNtech ProDiary, Philips Actiwatch Spectrum Plus, or Samsung Gear Life) [62].

EMAs have mostly been applied in eHealth and mHealth studies to measure health behavior and determinants (eg, [63,64]). We identified 1 study from previous reviews that used EMA to measure user engagement. This study [65] used event-based sampling to assess the breaks in levels of presence with a shooter game (not intended to improve health). The events that were sampled consisted of several parts of game play. No validity or reliability information for the slider was explicitly provided.

Despite the limited application of EMAs to measure engagement so far, EMAs may be well suited to study moment-to-moment or microlevel engagement with an intervention [5]. EMAs could provide data-driven insights into reasons for low adherence or dropout. EMAs are usually conducted over a short period with regular measurements over the day or week. However, it is also possible to adjust the timing and measurement intervals to collect longer-term insights into engagement. Contextual data and determinant data provided in EMA may enrich intervention usage data obtained from other sources to provide further insights into reasons for dropout.

#### Considerations

EMA surveys are intended to be very brief, because the purpose is to capture experiences in the moment and often to collect many data points over time, which can pose a burden to users [61]. Ensuring measures are brief is, therefore, important for both validity and for promoting adherence to the EMA protocol. Recent reviews of adherence to EMA protocols in health settings [66,67] suggest that compliance rates (proportion of EMAs completed) are reasonable (>70%), especially when sampling protocols are easy to follow. This speaks to the feasibility of utilizing this measurement approach; however, data analysis can be challenging for those unfamiliar with intensive longitudinal datasets (for a discussion regarding the challenges of EMA and example analysis approaches, see [68-72]). Advantages of EMAs include less recall bias than retrospective self-reports and potential for high ecological validity, as it studies behavior or effects in real-world contexts [60,61].

### System Usage Data

#### Focus Area

System usage data quantitatively capture how the intervention is physically used by each participant. This relates to the behavioral component of microlevel engagement. When paired with other data sources, system usage data can provide insights into how usage patterns, intervention dose, and different adherence rates relate to other aspects of engagement (eg, interest, attention, affect, and changes in determinants) and efficacy and effectiveness outcomes (eg, [73-76]).

#### Current Use and Future Directions

System usage data are the most commonly collected and reported measures of engagement in eHealth and mHealth interventions [4]. Although the focus has predominantly been on nonusage attrition and overall adherence to the intervention [3,8], more recent studies have begun to explore the multidimensional nature of usage data [77-79], focusing on the depth and type of engagement as well as frequency measures. As the field progresses, it would be helpful to have shared ways of conceptualizing these data, as recent reviews have tended to categorize types of usage data differently using an inductive approach [4,78]. The FITT acronym [80], which stands for frequency, intensity, time, and type, and is commonly used in physical activity research, might be a useful tool in this sense, especially for considering usage data as an engagement measure *a priori*. Specific examples of how usage data could be categorized using this principle are given in Table 3. Frequency provides information on how often a participant visits the intervention site or uses the app. Intensity measures the strength or depth of engagement with the intervention, for example, the proportion of the intervention site or app features used out of the total available features [4]. Type refers to the type of engagement, for example, this could be categorized as reflective (eg, self-reporting behavior change), altruistic (eg, helping others), or gamified (eg, participating in a challenge) in nature. Type can also be divided into “active” (eg, active input such as when responding to a quiz, self-monitoring, or writing an action plan) or “passive” (eg, an individual can view the intervention without having to interact with it) categories. Time is a measure of the duration of engagement during any single visit or a measure to assess level of exposure as an aggregate over the intervention period.

Examining usage data by aggregating data across the FITT categories can provide greater insights into engagement than focusing on any one domain [77,79,88]. For example, although the total time on site for users may appear similar (time data), their intensity data could be meaningfully different, which could lead to differences in engagement profiles (eg, attention, elaboration, and experience [79,88]). Separating users with similar data for time on site but markedly different patterns of use in terms of the type of activities may be helpful for identifying what aspects of the intervention are more engaging than others [92]; what aspects may be more influential for achieving behavior change, and in addition, whether this is moderated by user profiles (eg, [88]). The insight obtained from careful examination of system usage data in this way can assist intervention developers with data-driven solutions to encourage engagement [93].

**Table 3:** Examples of system usage data and type of information recorded.

Frequency, intensity, time, and type (FITT) principle		Example application
**Frequency of engagement with the intervention**		[81-83]
	Log-in (number of log-ins recorded per participant, average log-ins per unit of time or total for intervention duration)	
	Visits to the site (number of visits/hits per participant, average per unit of time or total)	
**Intensity of engagement**		[84-87]
	Pages viewed (number)	
	Lessons or modules viewed (total number, % of prescribed)	
	Posts viewed (eg, lurking)	
	Number of emails sent	
	Number of posts written	
	Accessed “Expert forum” (Ask the Expert) to pose a question/seek advice (number)	
	Action plan created	
	Number of quizzes attempted	
**Time or duration of engagement with the program**		[88,89]
	Amount of time spent at each visit per participant (average and total minutes)	
	Number of days between first and last log-in (duration or intervention *stickiness*)	
**Type of engagement**		[90,91]
	Reflective (eg, participant recording of behavior or health status)	
	Gamified (eg, accepting challenges and sending gifts)	
	Altruistic (eg, helping others) or malevolent (eg, trolling others)	
	Didactic (eg, reading posts and taking quizzes)	
	Active (eg, recording behavior) versus Passive (eg, reading posts).	

#### Considerations

User behavior in digital health interventions can be tracked by embedding programming code as part of the development process or by using third-party services. For both methods, it is important during software design (or selection) to consider the type of data desired or needed to track behavioral engagement and ensure the data are adequately captured and can be extracted easily. The most commonly used third-party service is Google Analytics, a service that can be implemented by connecting to the Google Analytics application programming interface. Google Analytics can be used to collect information on the users’ environment (location, browser, and connection speed), and the users’ behavior (eg, number of page visits, time on site, where users came from, and which page they visited last before exiting [94]). Capturing usage data more specific to the intervention platform, such as participation in a quiz or percentages of answers correct, require, as in Google Analytics, intentional programming and capture at the level of the software. Before programming, considerable thought should be given to how the usage data will be analyzed, as good tracking generates a large amount of data (ie, every navigational move that every participant has ever made and even the moves they did not make) that can be hard to make sense of; therefore, an *a priori* analysis plan is recommended. Visualization tools [82] and engagement indices such as those discussed by Baltierra et al [79] and Couper et al [88], or consideration of new data analyses techniques may be useful to get insights into data [95,96]. Although system usage data are often considered objective and reliable, some caution interpreting data is recommended. The increasing use of dynamic internet protocol (IP) addresses and virtual private networks (which change or hide your IP address), the use of IP addresses shared by multiple users (eg, via the family computer and internet cafes), and typical browsing behavior (eg, leaving multiple tabs open) may obscure usage data, especially for applications that do not require a unique log-in. This may be less of an issue for mobile apps compared with websites.

Intervention developers should, wherever possible, collect and analyze system usage data. Compared with the usage of other behavioral interventions (eg, a printed booklet), these data can be easily collected with early planning and good data capture techniques. Although usage data does not provide direct information on the psychological form of user engagement [4,5], it can provide some information to help us to understand what is engaging about an intervention, and what is not, in an unobtrusive way. There is also some evidence of predictive validity, with technology usage generally correlating with positive behavior change or health outcomes [81,91,97,98]. However, more research to establish the predictive validity of system usage data is needed, especially given that most analyses to date have lacked a suitable control group.

As with analyzing intensive longitudinal EMA data, the analysis of system usage data can be challenging. This is due to the intensive longitudinal and multidimensional nature of the data as well as the pattern of missingness (which tends to be nonrandom and nonignorable). Recognizing this, a comprehensive analysis plan should be developed before the commencement of the study. Exploration of the data visualization tools, composite engagement metrics, and analysis approaches referenced above might assist with the development of this plan.

It is also recommended that developers consider and outline the intended usage of the intervention. Intended usage is the way in which individuals should experience the intervention to derive maximum benefit, based on the conceptual framework informing intervention design (ie, developers’ views on how the intervention should work best for who). Notably, intended usage may not be the same for all individuals (eg, in adaptive interventions [99,100]). By specifying intended usage *a priori* and comparing this with observed usage, we can establish whether individuals have adhered to the intervention and, in turn, the impact of adherence on efficacy [3].

### Sensor Data

#### Focus Area

Sensors such as global positioning systems (GPS), cameras (eg, facilitating eye tracking analyses), microphones, and accelerometers can unobtrusively monitor users’ behavior and the physical context in which this behavior takes place. They can be provided by the investigator, but many of them are embedded in smartphones or trackers. This relates to the behavioral component of microlevel (eg, information on intervention fidelity) and macrolevel (eg, tracking behavior in real-life settings) engagement.

#### Current Use and Future Directions

Analyzing sensor data presents an unobtrusive way of measuring engagement that requires no additional time effort from users other than the time spent engaging with the program. Their value lies in being able to track behavior of many users [101] and to enrich usage information in real-life situations or combining them with other user engagement measures such as EMA. There are calls for a different evaluation of eHealth and mHealth behavior change interventions than traditional interventions, to more nimbly respond to rapidly changing technologies and user preferences for functionalities [102-105]. Adaptations to eHealth and mHealth interventions are likely to be needed soon after first design and again after first implementation. Information from sensors that automatically track usage in real-life situations can help in measuring engagement with these interventions and distinguishing between successful mastery of intervention goals or need for continued engagement [5]. For example, in physical activity interventions, accelerometer information could continuously monitor the current activity level and indicate whether lower adherence to the intervention should be considered as a successful completion or disengagement. Sensor data paired with usage information may thus provide insights in macrolevel engagement as a mediator of positive intervention outcomes. In a similar vein, GPS information can enrich macrolevel engagement measures. GPS gives information on where people use the intervention and where it is less often used. For example, an app designed to facilitate healthy food choices may be used at home or at grocery stores but shows lower usage in restaurants. The GPS data give further insight into offline engagement with the intervention goals.

Sensors can also provide an indication of intervention fidelity. For example, distance traveled as measured by GPS and phone cameras taking pictures of meals can indicate whether the intervention is used in the appropriate manner and context [106]. The combination of usage and commonly included sensors can provide more detailed measures of real-life user engagement than usage information by itself. Sensor data can, moreover, trigger the event-based form of EMA. For example, users may be prompted to indicate their engagement with the intervention when the accelerometer shows the person is physically inactive or assess user engagement when GPS data show the person is in a certain physical context (eg, at a bar where there is a personal risk of smoking or alcohol consumption).

#### Considerations

A challenge of using GPS data for this purpose is the time-intensive nature of GPS data preparation and analysis. This will likely get easier in the future as new analysis packages become available to facilitate automation. Sensors, moreover, have the advantage of presenting a low level of respondent burden. However, especially with context-aware sensing using GPS, users are concerned about privacy issues [107,108]. In addition, sensors integrated in smartphones tend to negatively impact the battery life of the mobile device, and users may, therefore, be less compliant with running these sensors on their phones. This may especially be the case when users are skeptical toward the accuracy and relevance of context-aware smartphone sensing [25]. Therefore, communicating research findings about the validity of such measures [109,110]) and conducting pilot tests and validity studies of new measures may be necessary to increase their use in future interventions and optimize uptake among participants.

### Social Media

#### Focus Area

Another unobtrusive, low-burden approach to capturing engagement with the intervention is to analyze users’ social media patterns. In social media, users create online communities (eg, social networking sites) via which they share information, opinions, personal messages, or visual material. Despite the interest of behavior change professionals in using social media to increase intervention effectiveness (see eg, [111]), to our knowledge, little research is available on the use of social media to measure engagement with eHealth and mHealth. The available resources mostly come from marketing and media audience research [112,113]. Social media message threads may provide useful information on user experience (microlevel engagement with the intervention) but might also provide insights in macrolevel engagement (eg, wall posts on behavioral achievements).

#### Current Use and Future Directions

One study examined the number of wall posts made over time as an indication of engagement with a social networking physical activity intervention [114]. An approach to reduce the burden in analysis is to use *markers* that are previously nonexisting words launched exclusively within the intervention [115]. These markers are used to trace any conversation that takes place on social media in relation to the intervention and are a way to measure social proliferation associated with the intervention content. An example comes from a video intervention on cognitive problems that may result from being a victim of violence [115]. To clearly identify all conversations and mentions on social media that would result from this topic, they launched the word *falterhead* to describe how the main character experienced the negative effects on his brain functioning after being violently attacked. This marker allowed a quick identification of all social media content related to the program, as this nonexisting word is unlikely to occur for content unrelated to the intervention. Several social media sources are then searched with text- and data-mining tools (eg, HowardsHome Finchline) for the occurrence and content of messages that contain these markers. The messages are next analyzed in terms of quantity (eg, Is the intervention being talked about?; What are patterns of social proliferation over time?) and quality (eg, How is the topic mentioned or discussed?; Is this how we wished viewers would think and talk about the intervention?). Social media messages relating to the eHealth and mHealth intervention might also be analyzed for their occurrence of certain profiles in social media engagement. On a continuum from passive and uninterested to more active and engaged, profiles of lurkers, casuals, actives, committed, and loyalists can be distinguished. Although to our knowledge, this has not yet been applied to analyze engagement with eHealth and mHealth behavior change interventions, interventions showing more actives, committed, and loyalists on social media might indicate higher user engagement than those receiving more lurkers and casuals [116]. This might especially be useful to assess comments on engagement in behavior change programs in real-life settings.

#### Considerations

The vast amount of social media content may make it difficult to extract what is relevant to the intervention. Markers mentioned earlier and audit tools are useful to facilitate such social media analyses. Examples of free audit tools to analyze social media are Sprout Social Simply Measured, Instagram Insights, and Union Metrics. The free statistical software program R also has many packages to analyze social media data. The analysis of these social media patterns requires a combination of qualitative techniques to assess discussion or post sentiment and topic, and quantitative methods, for example, to assess reach by combining number of followers for each mention on social media [117]. Text analytic tools available in many statistical packages such as R and SAS may also be useful here.

### Psychophysiological Measures

#### Focus Area

Psychophysiological methods of measurement are used to examine the relationship between physiology and overt behavior or cognitive processes and variables. Psychophysiological measures are operationalization of cognitive processes or variables, just as self-reported questionnaires are used to measure processes or variables derived from theory [118]. They have been shown to be valuable approaches for measuring the experiential aspects of microengagement [119].

#### Current Use and Future Directions

There are several types of psychophysiological measures used to study cognitive and affective processes (for a comprehensive overview of measures used in human-computer interaction and user experience research, see [119-121]). We describe the 2 most common methods with a strong temporal resolution (ie, electroencephalography [EEG] and eye-tracking). A strong temporal solution (ie, precision of measurement with respect to time) is warranted to investigate engagement over time. It needs to be stressed, however, that other methods show promising results as well [122-127]. For example, predicting engagement using a novel visual analysis approach to recognize affect performed significantly better or on par with using self-reports [125]. The methods presented here are noninvasive but obtrusive in comparison with, for example, most measurements of system usage data. These methods are mostly used in laboratory settings and during intervention development (eg, pretesting of a website), but the opportunities to use them in field settings are increasing (eg, [128]). Moreover, it is also possible to use these methods in parallel with a trial or afterward to gain more insight into user engagement and, thereby, shed more light on trial findings.

EEG records electrical activity in the brain using small, flat metal discs (electrodes) attached to a person’s scalp. Using this method requires adequate expertise, both in terms of measurement [129] and analysis [130] of data. Event-related potentials (ERPs) are the *average* changes in the EEG signal in response to a stimulus, and characteristic ERP responses are referred to as components [131]. For example, Leiker et al [132], in a study on motion-controlled video games, focused on the amplitude of a specific component (labeled eP3a), which is a reliable index of attentional reserve [133,134]. This study revealed that participants who reported higher levels of engagement (as measured by the Intrinsic Motivation Inventory) showed a smaller eP3a, which is indicative of paying more attention to the primary task (eg, playing the game). Another study revealed that late negative slow wave components of the ERP were indicative of attention, which was partly confirmed by findings from self-reports (ie, the Immersive Experience Questionnaire) [123].

Eye-tracking is based on the strong association between eye movements and attention [135]. It is a suitable method to assess the course of attention over time [136]. For example, fixation data of an experimental study revealed that participants’ eye movements in the immersive condition decreased over time, which is indicative of increased attention [137]. Another example is a study comparing a video with a text condition of a physical activity intervention. This study revealed that participants in the video condition displayed greater attention to the physical activity feedback in terms of gaze duration, total fixation duration, and focusing on feedback [138]. Another study using eye-tracking found that participants focused more on certain experimentally manipulated aspects of a health-related website (ie, in terms of frequency and duration), but this did not affect usage data (ie, the number of pages visited or the time on the website) [139]. It might be that these aspects attract attention, but there is a trade-off in the sense that participants then focus less on other aspects of the website. However, it could also be that attention only partly predicts engagement.

#### Considerations

With regard to both EEG and eye-tracking, it is important to note that attention is only the first appraisal in the process of engagement [139]. There are other psychophysiological methods besides EEG and eye-tracking that are mostly focused on measuring arousal. A previous study, for example, recorded electrodermal activity (EDA) and facial muscle activity (electromyography [EMG]) in addition to a Game Experience Questionnaire [140]. The association between these measures, however, was not straightforward. For example, EMG orbicularis oculi (periocular) is usually used to indicate positive emotions and high arousal but was negatively correlated to competence (which is a positive dimension of the Game Experience Questionnaire). Another study measured engagement in 5 different ways: self-reports using 4 dimensions of the Temple Presence Inventory, content analyses of user videos, EDA, mouse movements, and click logs (the latter 2 are measurements of usage data) [124]. These 5 measures correlated in limited ways. The authors concluded that “engagements as a construct is more complex than is captured in any of these measures individually and that using multiple methods to assess engagement can illuminate aspects of engagement not detectable by a single method of measurement” [124].

This is indicative of the complexity of engagement as a construct and reflects recent calls from the human-computer interaction field for future studies to identify valid combinations of psychophysiological measures that more fully capture the multidimensional nature of engagement [119].

## Discussion

It is generally agreed that some form of engagement is necessary for eHealth and mHealth behavior change interventions to be effective. However, cohesive and in-depth knowledge about how to develop engaging interventions and the pathways between engagement and efficacy are lacking. Several models of engagement have been proposed in the literature to address this deficit, but little testing of the models has been conducted. To support research in this area and progress the science of user engagement, we aimed to provide a comprehensive overview of the measurement options available to assess engagement in an eHealth and mHealth behavioral intervention setting. The overview should not be treated as exhaustive; however, it should serve as a useful point of reference when considering engagement measures for behavioral eHealth and mHealth research.

The best measurement approach will likely depend on the stage of research and the specific research context, although there are benefits from using multiple methods and pairing the data (eg, self-report data relating to interest, attention and affect combined with system usage data). It is also important to make an inventory—before data collection—to check whether the available expertise for using different methods (eg, EEG) is available. Given the complexity of engagement as a construct, using multiple methods may be necessary to illuminate it fully [119,124]. At present, most studies in the eHealth and mHealth behavioral intervention space rely on system usage data only. Although system usage data is undoubtedly a valuable engagement marker, it is not considered a valid measure of micro- or macroengagement on its own [4,5]. Greater efforts are needed to also assess the psychological aspects of engagement to better understand the interplay between perceptions, usage, and efficacy.

Questionnaires are perhaps the most accessible way to assess microlevel engagement in terms of cost. However, there is currently a lack of validated self-report questionnaires specific to the eHealth and mHealth behavior change intervention context. This is reflected in the large number of purpose-built questionnaires (ie, questionnaires designed for a specific study) that have been used to date [4]. As the main benefit of questionnaires is that they allow for the collection of subjective data in a standardized way, greater efforts are needed to develop and implement standard items. Although not yet validated, the questionnaire developed by Perski et al [44] is promising in this regard, as it includes constructs related to both psychological and behavioral aspects of engagement and only focuses on engagement constructs. The other questionnaires identified focus only on the psychological aspects of engagement, and some include constructs more aligned with standard acceptability items (eg, perceived credibility), rather than the constructs of interest, attention, and affect. It may be best to avoid these questionnaires when testing models that hypothesize that acceptability markers influence engagement parameters.

There are several other measures of engagement that may also be used to test engagement models (eg, sensors, social media data, EMA, and psychophysiological measures). Despite their potential advantages, little research has been conducted exploring their use (and validity) in the digital behavior change setting. This is likely due to higher cost, time, and data analysis requirements relative to other measures. To mitigate this, behavioral researchers are increasingly drawing on expertise across other relevant disciplines (eg, informatics, human-computer interaction, experimental, and cognitive psychology). It is hoped that this paper will help to facilitate this research, especially research establishing the criterion, as well as divergent and predictive validity of these measures.

Overall, establishing the validity of engagement measures across multiple settings and learning how to triangulate measures in a complementary way are necessary next steps to advance the field. This will allow us to thoroughly test contemporary models of user engagement and hence, deepen our understanding of the interplay between intervention perceptions, usage, and efficacy across different settings.

## Appendix

Multimedia Appendix 1 Initial data extraction table.

Multimedia Appendix 2 Self-report questionnaires for measuring microlevel engagement.

Multimedia Appendix 3 Example items in self-report questionnaires for measuring microlevel engagement.

## Abbreviations

EDA: electrodermal activity
EEG: electroencephalography
eHealth: electronic health
EMA: ecological momentary assessment
EMG: electromyography
ERP: event-related potentials
FITT: frequency, intensity, time, and type
IP: internet protocol
mHealth: mobile health
NHMRC: National Health and Medical Research Council
