# Supplementary material for: Measuring Engagement in eHealth and mHealth Behavior Change Interventions: Viewpoint of Methodologies
Source: J Med Internet Res. 2018 Nov 16;20(11):e292. doi: 10.2196/jmir.9397 (PMC6269627; doi:10.2196/jmir.9397)
Supplement: Multimedia Appendix 2 [file jmir_v20i11e292_app2.pdf]

Multimedia Appendix 2. Self-report questionnaires for measuring micro-level engagement

**Measure**	**Constructs**	**Description**	**Scoring**	**Psychometric Properties**	**Example Application**
**The User Engagement Scale** [1, 2]	Overall evaluation of user experience, assessing six attributes: Focused attention, Perceived usability, Aesthetics appeal, Endurability, Novelty, Felt involvement.	31-item scale; items measured on a 5-point Likert scale ranging from 1 (strongly disagree) to 5 (strongly agree).<br><br>Adaptation of items would be needed for health setting. Re-analysis used a four-factor solution where Endurability, Novelty, Felt involvement were grouped into one factor (Reward factor).	Negatively worded items are reverse scored. Items are then summed to create an overall score; subscale scores can also be created by summing relevant items. Higher scores indicate higher engagement.	Developed in an e-commerce setting.<br><br>Original study confirmed 6 distinct factors, with alpha for each factor reasonable [1]. However, the subscales have not been stable across research settings [2].<br><br>Predictive validity: In a study that applied the scale to video games and found a 4-factor solution, the scale was more predictive of game performance than the Flow State Scale [3].<br><br>Contains attributes hypothesised to influence engagement (e.g., aesthetic appeal), as well as attributes of engagement (e.g., focused attention). Re-analysis of the original data	[5-7]

failed to produce a good structure rot the six-factor structure, but showed a good model for the four-factor structure. But only focused attention, perceived usability and aesthetics appeal were coherent factors [4].

**User Engagement Scale - Short form [4]**	Short form of the User engagement scale assessing Focused attention, Perceived usability, Aesthetics appeal, reward factor.	12- items scale; items measured on a 5-point Likert scale ranging from 1 (strongly disagree) to 5 (strongly agree).	Negatively worded items are reverse scored. Items are then summed and divided by twelve to create an overall score; subscale scores can also be created by summing the three items and divide by three. Higher scores indicate higher engagement.	Developed in a e-commerce setting and validated on the social book search data. A good internal reliability was seen for all three subscales ranging from 0.70-0.88).	None found.

**eHealth Engagement Scale [8]**.	4 sub-scales: Involving (4 items: absorbing, attention-grabbing, stimulating, surprising), credible (3 items: convincing, balanced, believable), negative feelings (one item: not dull), amusing/friendly (one item: hip/cool).	9-item scale; Items measured on a 5-point Likert scale, ranging from 1 (strongly agree) to 5 (strongly disagree).	Higher scores indicate higher engagement. Detailed information about the scoring instructions is not publically available. Email author for scoring instructions.	Adapted from a questionnaire designed to evaluate television watching.<br>A 12-item questionnaire was originally tested and results from an exploratory factor analysis suggested a two-factor solution: Involving (assessing the extent that the intervention was perceived as absorbing, attention-grabbing and surprising) and stimulating (assessing the extent the intervention was perceived as suspenseful, clever and hip/cool). Internal reliability of the two subscales was 0.87 for involving and 0.81 for credible [8].<br><br>A four-factor solution, consisting of 9-items was found to be superior in-terms of predictive validity. The four factors together (9-item	None found.

**Digital Behaviour Change Intervention Engagement Scale** (version 1) [9]	Interest, attention, enjoyment, amount of intervention use, and depth of intervention use.	10-item scale: majority (9/10) of items measured on a 7-point Likert scale with endpoints and middle anchored: not at all; moderately; extremely. Final item lists intervention components and asked which components the user recalls visiting.	Higher scores indicate higher engagement.<br><br>Detailed information about the scoring instructions is not publically available. Email author for scoring instructions.	A detailed protocol for testing the reliability and validity (construct, criterion and predictive) of the scale have been published [39]. The scale is currently being tested in an alcohol reduction study, involving the smartphone app "*Drink Less*". More information, including a copy of the scale is available via the studies open science framework page [9].	[9].

**User Experience Questionnaire** [10]	6 sub-scales: attractiveness, perspicuity, efficiency, dependability, stimulations, and novelty.	26-item scale; items measured on a bipolar Likert scale. The items are scaled from -3 to +3. -3 represents the most negative answer, 0 a neutral answer, and +3 the most positive answer.	Negatively worded items are reverse scored. Items are then averaged to create subscale scores. Higher scores equate to higher engagement/better user experience. Standard interpretation: -0.8 to 0.8 represents a neutral evaluation; values > 0.8 represent a positive evaluation and values < 0.8 represent a negative evaluation.	Initially designed to examine a product's ability to promote an engaging user experience. The raw-version of the questionnaire containing 80 items assessing attractiveness and the quality of the product was tested in six sub studies. The data was split into two data sets; data set 1 contained 14 items and data set 2 contained 66 items. A one-factor solution was found for data set 1 (explained 60% of the observed variance) and the six items with the highest factor loadings were selected. A five-factor solution was found for data set 2 (explained 53% percent of the observed variance). To reduce the number of items, 4 items were selected per factor. A second factor analyse on the reduced data set 2 extracted five	[11, 12]

**Enjoyment of website experiences scale** [13]	3 sub-scales: Engagement (focused attention), Positive affect, Fulfilment.	12-item scale; items measured on a 7-point Likert scale, ranging from strongly agree to strongly disagree	Subscale scores can be created by adding sub-scale items together, or by computing item average. Overall enjoyment score can also be computed by adding or averaging all items. Recommend to code so that higher scores indicate a more positive user experience.	Tests were conducted in a student population, using two websites. Exploratory factor analyses were conducted to examine the 14 items of the questionnaire. A three-factor structure (87.5% of the total variance) was found superior to a single factor structure (77.3% of the total variance). Based on the feedback of the participants two items were removed from the questionnaire and the scale was modified from a 9-point scale to a 7-point scale. A high and stable reliability was found (Cronbach's Alpha >0.9). The internal validity of the confirmatory model was reasonably acceptable (Root Mean square Residual=0.08, Adjusted	[14]

Goodness of Fit Index=0.76, CFI=0.97)[13].

**Cognitive Absorption scale** [15]	5 sub-scales: Temporal dissociation, Focused immersion, Heightened enjoyment, Control, Curiosity	20 item scale; items measured on a 7-point scale (1 - strongly disagree to 7 strongly agree).	Sub-scale scores created by averaging relevant items. Higher scores indicate higher engagement.	Reliability and discriminant validity were examined in a student sample. Satisfactory reliability (factor loading for (>0.7) all items except for three items and composite reliability ≥0.88) Satisfactory discriminant validity (determined by confirmatory factor analysis); all indicator load more highly on their own construct than other constructs and all constructs share more variance with their indicators than with other constructs [15].	[68]

**Gaming Engagement Questionnaire [16]**	4 sub-scales: Absorption, Flow, Presence, Immersion	19 positively worded questions answered on a seven-point Likert scale: the higher the score the user gives, the more engaged they are.	Subscale scores can be created by adding sub-scale items together. An overall engagement score can also be computed by adding all items.”	Initially developed to assess impact of deep engagement in violent video games. Based on existing approaches to measuring presence, flow, absorption, and dissociation.<br>In the first phase of development, selected items based on previous measures were discussed in focus groups among child and adult video game players. 15-item pilot versions were then administered to two different samples. A Rasch rating scale analysis suggested that additional items were needed to cover the engagement level of respondents. An additional 4 items were added and the scale was re-tested [16].<br><br>The 19-item scale was administered to a 153 high school students.	[17]

**Immersive Experience Questionnaire** [18]	5 sub-scales: Cognitive Involvement, Real World Dissociation, Challenge, Emotional Involvement, Control	31-item scale. Items measured on a five-point Likert scale ranging from strongly disagree to strongly agree. There are 6 negatively worded items.	Negatively worded items are reverse scores. Immersion scores are calculated by summing scores for all 31 items.	Used across a diverse range of game genres. Principal component analysis identified five main factors (Cognitive Involvement, Real World Dissociation, Challenge, Emotional Involvement and Control), accounting for 49% of the variance. Authors concluded the questionnaire measures a mixture of person factors (cognitive involvement, real world dissociation, emotional involvement) and game factors (challenge, control), and is measuring the same underlying factor, i.e., cognitive involvement, real word dissociation, emotional involvement, challenge and control.<br>In practice, immersion is	[19]

often treated as a single dimension with the factors used to aid in the interpretation of results[18].

References

1. O'Brien, H.L. and E.G. Toms, *The development and evaluation of a survey to measure user engagement.* Journal of the American Society for Information Science and Technology, 2010. **61**(1): p. 50-69.
2. O’Brien, H.L. and E.G. Toms, *Examining the generalizability of the User Engagement Scale (UES) in exploratory search.* Information Processing & Management, 2013. **49**(5): p. 1092-1107.
3. Jackson, S.A. and H.W. Marsh, *Development and Validation of a Scale to Measure Optimal Experience: The Flow State Scale.* Journal of Sport and Exercise Psychology, 1996. **18**(1): p. 17-35.
4. O’Brien, H.L., P. Cairns, and M. Hall, *A practical approach to measuring user engagement with the refined user engagement scale (UES) and new UES short form.* International Journal of Human-Computer Studies, 2018. **112**: p. 28-39.
5. Cian, L., A. Krishna, and R.S. Elder, *This Logo Moves Me: Dynamic Imagery from Static Images.* Journal of Marketing Research, 2014. **51**(2): p. 184-197.
6. Wiebe, E.N., et al., *Measuring engagement in video game-based environments: Investigation of the User Engagement Scale.* Computers in Human Behavior, 2014. **32**: p. 123-132.
7. Banhawi, F., N.M. Ali, and J. Hairuliza Mohd. *Measuring user engagement levels in social networking application*. in *Proceedings of the 2011 International Conference on Electrical Engineering and Informatics*. 2011.
8. Craig Lefebvre, R., et al., *The Assessment of User Engagement with eHealth Content: The eHealth Engagement Scale.* Journal of Computer-Mediated Communication, 2010. **15**(4): p. 666-681.
9. Perski, O., et al., *Study protocol: Development and psychometric evaluation of a self-report instrument to measure engagement with digital behaviour change interventions*. 2017: 2018-07-13. URL:https://osf.io/cj9y7/ Accessed: 2018-07-13. (Archived by WebCite® at http://www.webcitation.org/70sBGZe9w)
10. Laugwitz, B., T. Held, and M. Schrepp, *Construction and Evaluation of a User Experience Questionnaire*, in *HCI and Usability for Education and Work: 4th Symposium of the Workgroup Human-Computer Interaction and Usability Engineering of the Austrian Computer Society, USAB 2008, Graz, Austria, November 20-21, 2008. Proceedings*, A. Holzinger, Editor. 2008, Springer Berlin Heidelberg: Berlin, Heidelberg. p. 63-76.
11. Morris, R.R., S.M. Schueller, and R.W. Picard, *Efficacy of a Web-Based, Crowdsourced Peer-To-Peer Cognitive Reappraisal Platform for Depression: Randomized Controlled Trial.* J Med Internet Res, 2015. **17**(3): p. e72.
12. Schrepp, M., A. Hinderks, and J. Thomaschewski, *Applying the User Experience Questionnaire (UEQ) in Different Evaluation Scenarios*, in *Design, User Experience, and Usability. Theories, Methods, and Tools for Designing the User Experience: Third International Conference, DUXU 2014, Held as Part of HCI International 2014, Heraklion, Crete, Greece, June 22-27, 2014, Proceedings, Part I,* A. Marcus, Editor. 2014, Springer International Publishing: Cham. p. 383-392.
13. Lin, A., S. Gregor, and M. Ewing, *Developing a scale to measure the enjoyment of Web experiences.* Journal of Interactive Marketing, 2008. **22**(4): p. 40-57.
14. Lustria, M.L.A., et al., *A model of tailoring effects: A randomized controlled trial examining the mechanisms of tailoring in a web-based STD screening intervention.* Health Psychology, 2016. **35**(11): p. 1214-1224.

15. Agarwal, R. and E. Karahanna, *Time Flies When You're Having Fun: Cognitive Absorption and Beliefs about Information Technology Usage.* MIS Quarterly, 2000. **24**(4): p. 665-694.
16. Brockmyer, J.H., et al., *The development of the Game Engagement Questionnaire: A measure of engagement in video game-playing.* Journal of Experimental Social Psychology, 2009. **45**(4): p. 624-634.
17. Smith, L.J., et al., *Intrinsic and extrinsic predictors of video-gaming behaviour and adolescent bedtimes: the relationship between flow states, self-perceived risk-taking, device accessibility, parental regulation of media and bedtime.* Sleep Medicine, 2017. **30**: p. 64-70.
18. Jennett, C., et al., *Measuring and defining the experience of immersion in games.* International Journal of Human-Computer Studies, 2008. **66**(9): p. 641-661.
19. Cairns, P., et al., *Who but not where: The effect of social play on immersion in digital games.* International Journal of Human-Computer Studies, 2013. **71**(11): p. 1069-1077.
