# Supplementary material for: Measuring Engagement in eHealth and mHealth Behavior Change Interventions: Viewpoint of Methodologies
Source: J Med Internet Res. 2018 Nov 16;20(11):e292. doi: 10.2196/jmir.9397 (PMC6269627; doi:10.2196/jmir.9397)
Supplement: Multimedia Appendix 3 [file jmir_v20i11e292_app3.pdf]

Supplementary Table 2 - Example items in questionnaires

Measure	Example items
**The User Engagement Scale** {O'Brien, 2010 #3688;O'Brien, 2013 #3977}	**Focused attention**<br>'I lost my-self in this shopping experience'<br>'I blocked out the things around me when I was shopping on this website.'<br><br>**Perceived usability**<br>'I felt frustrated while visiting the shopping website.'*<br>'I felt discouraged while shopping on this website.'*<br><br>**Aesthetics appeal**<br>'The shopping website is attractive.'<br>'The shopping website was aesthetically appealing'<br><br>**Endurability**<br>'Shopping on this website was worthwhile'<br>'This shopping experience did not work out the way I had planned'*<br><br>**Novelty**<br>'I continued to shop on this website out of curiosity'<br>'I felt interested in this shopping task'<br><br>**Felt involvement**<br>'I was really drawn into my shopping task'<br>I felt involved in this shopping task'<br><br>**5-point likert scale, ranging from strongly disagree to strongly agree*
**eHealth Engagement Scale** {Craig Lefebvre, 2010 #5176}.	**Involving**<br>'Absorbing'<br>'Attention-Grabbing'<br>'Surprising'

	**Credible**<br>'Convincing'<br>'Balanced'<br><br>**Negative feelings**<br>'Not dull'<br><br>**Amusing/Friendly**<br>'Hip/Cool'<br><br>**5-point Likert scale, ranging from strongly agree to strongly disagree.*
**DBCI Engagement Scale** (version 1) {Perski, 2017 #6336} **https://osf.io/cj9y7/**	**Interest/enjoyment/attention**<br>'How strongly did you experience the following?'<br>'Interest'<br>'Focus'<br>'Enjoyment'<br>**7-point likert scale, ranging from not at all to extremely*<br><br>**Amount of use**<br>'How much time (in minutes) do you roughly think that you spent on the app?'<br>*free text<br>**Depth of use**<br>'Which of the app's components do you remember visiting?'<br>**components listed. Depth is indexed as a percentage of available components.*
**User Experience Questionnaire** {Laugwitz, 2008 #6232} *Questionnaire and*	**Attractiveness**<br>'Annoying/ enjoyable'<br>'Unlikeable/ pleasing'<br>**Perspicuity**<br>'Easy to learn/ difficult to learn'

handbook downloadable from http://www.ueq-online.org/	'Not understandable/ understandable'<br><br>**Efficiency**<br>'Fast/slow'<br>'Organised/ cluttered'<br><br>**Dependability**<br>'Unpredictable/ Predictable'<br>'Secure/Not secure'<br><br>**Stimulations**<br>'Boring/ Exciting'<br>'Not Interesting/ Interesting'<br><br>**Novelty**<br>'Creative/Dull'<br>'Inventive/ conventional'<br>**Bi-polar likert scale from -3 to +3.*
**Enjoyment of website experiences scale** {Lin, 2008 #3644}	**Engagement (focused attention)**<br>'I was deeply engrossed'<br>'My attention was focused'<br>**9-point likert scale from strongly agree to strongly disagree*<br><br>**Positive affect**<br>'Happy/unhappy'<br>'Relaxed/bored'<br>**9-point semantic differential scale*<br><br>**Fulfilment (Need or Desire)**<br>'It meant a lot to me'<br>'It was useful'<br>**9-point likert scale from strongly agree to strongly disagree*
**Cognitive**	**Temporal dissociation**

**Absorption scale** {Agarwal, 2000 #6241}	‘Time appears to go by very quickly when I am using the Web’<br>‘Most times when I get on to the Web, I end up spending more time that I had planned’<br><br>**Focused Immersion**<br>‘While I am using the Web I am able to block out most other distractions’<br>‘While on the Web, I am immersed in the task I am performing’<br><br>**Heightened enjoyment**<br>‘I have fun interacting with the Web’<br>Using the Web bores me’<br>**7-point likert scale from strongly disagree to strongly agree*
**Gaming Engagement Questionnaire** {Brockmyer, 2009 #6242}	**Absorption**<br>‘I lost track of where I am.’<br><br>**Flow**<br>‘If someone talks to me I don’t hear’<br>‘I can’t tell I’m getting tired’<br><br>**Presence**<br>‘Things seem to happen automatically’<br>‘The game feels real’<br><br>**Immersion**<br>‘I really get into the game’<br>**7-point likert scale*
**Immersive Experience Questionnaire** {Jennett, 2008 #6246}	**Basic attention**<br>‘To what extent did the game hold your attention?’<br>‘To what extent do you feel you were focused on the game?’<br><br>**Temporal dissociation**<br>‘To what extent did you lose track of time?’

	‘To what extent did you forget about your everyday concerns?’<br><br>**Transportation**<br>‘To what extent did you feel that the game was something you were experiencing, rather than something you were just Doing?’<br>‘To what extent was your sense of being in the game environment stronger than your sense of being in the real world?’<br><br>**Challenge**<br>‘To what extent did you find the game challenging?’<br>‘Were there any times during the game in which you just wanted to give up?’<br><br>**Emotional involvement**<br>‘Did you care if you won or lost?’<br>‘To what extent did you feel emotionally attached to the Game?’<br><br>**Enjoyment**<br>‘Did you like the task?’<br>‘How much would you say you enjoyed playing the game?’<br>**5-point likert scale, ranging from strongly disagree to strongly agree.*<br>*Nb. items were designed to assess the six constructs above. Factor analysis suggested five constructs, which the authors renamed.*
